# Cross-Effects in Folding and Phase Transitions of hnRNP A1 and C9Orf72 RNA G4 In Vitro

**DOI:** 10.3390/molecules29184369

**Published:** 2024-09-14

**Authors:** Tatiana Vedekhina, Julia Svetlova, Iuliia Pavlova, Nikolay Barinov, Sabina Alieva, Elizaveta Malakhova, Pavel Rubtsov, Alina Shtork, Dmitry Klinov, Anna Varizhuk

**Affiliations:** 1 Lopukhin Federal Research and Clinical Center of Physical-Chemical Medicine of Federal Medical Biological Agency, Malaya Pirogovskaya, 1a, 119435 Moscow, Russia; j.i.svetlova@gmail.com (J.S.); pavlova.yuiv@gmail.com (I.P.); nik_mipt@mail.ru (N.B.); stadashi6@gmail.com (S.A.); liza090871@gmail.com (E.M.); pavel.rubtcov11@gmail.com (P.R.); a.s.shtork@gmail.com (A.S.); klinov.dmitry@mail.ru (D.K.); 2 Center for Precision Genome Editing and Genetic Technologies for Biomedicine, Lopukhin Federal Research and Clinical Center of Physical-Chemical Medicine of Federal Medical Biological Agency, Malaya Pirogovskaya, 1a, 119435 Moscow, Russia; 3 Lomonosov Institute of Fine Chemical Technologies, MIREA–Russian Technological University, Vernadsky Avenue, 86, 119454 Moscow, Russia; 4 A.P. Nelyubin Institute of Pharmacy, I.M. Sechenov First Moscow State Medical University of the Ministry of Healthcare of the Russian Federation (Sechenov University), Trubetskaya Str., 8-2, 119991 Moscow, Russia

**Keywords:** liquid-liquid phase separation, biomolecular condensate, G4 RNAs, *C9Orf72*, RNA binding protein, hnRNP A1, SRSF, post-translational modifications, neurodegenerative diseases

## Abstract

Abnormal intracellular phase transitions in mutant hnRNP A1 may underlie the development of several neurodegenerative diseases. The risk of these diseases increases upon *C9Orf72* repeat expansion and the accumulation of the corresponding G-quadruplex (G4)-forming RNA, but the link between this RNA and the disruption of hnRNP A1 homeostasis has not been fully explored so far. Our aim was to clarify the mutual effects of hnRNP A1 and C9Orf72 G4 in vitro. Using various optical methods and atomic force microscopy, we investigated the influence of the G4 on the formation of cross-beta fibrils by the mutant prion-like domain (PLD) of hnRNP A1 and on the co-separation of the non-mutant protein with a typical SR-rich fragment of a splicing factor (SRSF), which normally drives the assembly of nuclear speckles. The G4 was shown to act in a holdase-like manner, i.e., to restrict the fibrillation of the hnRNP A1 PLD, presumably through interactions with the PLD-flanking RGG motif. These interactions resulted in partial unwinding of the G4, suggesting a helicase-like activity of hnRNP A1 RGG. At the same time, the G4 was shown to disrupt hnRNP A1 co-separation with SRSF, suggesting its possible contribution to pathology through interference with splicing regulation.

## 1. Introduction

The pathogenesis of a subset of neurodegenerative diseases referred to as proteinopathies is distinguished by abnormalities in protein post-translational modifications (PTM), folding, nucleoplasm-cytoplasm shuttling, phase separation, or degradation [1]. Aberrant phase separation, initiated by mutations, protein misfolding, PTM deregulation, or intracellular protein redistribution, frequently results in sequestration of the protein in liquid condensates or solid aggregates. This may eventually culminate in loss-of-function toxicity [2]. Furthermore, proteolysis-resistant solid aggregates of misfolded proteins can impede intracellular transport, particularly in neurons, thereby conferring additional (gain-of-function) toxicity [3].

Heterogenous nuclear ribonucleoproteins (hnRNPs) are among the most commonly dysregulated proteins in neuropathology, along with alpha-synuclein and amyloid-beta precursor proteins [4]. All hnRNPs contain at least one RNA recognition motif (RRM), which enables their sequence-specific binding to pre-mRNA and fine-tuning of pre-mRNA processing, including alternative splicing [5,6]. The RRM is typically flanked by a low complexity domain (LCD), which enables hnRNP packaging into biomolecular condensates with other splicing factors, mostly SR-rich ones (SRSFs), through liquid-liquid phase separation (LLPS) [7]. Such condensates are known as nuclear speckles [8]. They serve to “buffer” hnRNP and SRSF concentrations in the nucleoplasm and may directly participate in splicing [9].

HnRNP A1, one of the ubiquitously expressed hnRNPs, functions as a repressor of spliceosome assembly at weak splicing sites [10]. The disruption of its homeostasis has been linked to amyotrophic lateral sclerosis (ALS) and frontotemporal dementia (FTD) [11,12] and is considered a major cause of mis-splicing events associated with spinal muscular atrophy, multiple sclerosis, and Alzheimer’s disease [13,14,15,16,17]. One notable example of an affected gene is *MAPT*, which encodes the microtubule-associated protein tau [18]. This protein is normally present in two isoforms, differing in their affinity for tubulin, which allows for a dynamic equilibrium between microtubule assembly and disassembly. Imbalances in tau isoform production shift this equilibrium, disrupt microtubule-dependent intracellular transport, and potentially lead to tau aggregation. Thus, there appears to be a mechanistic link between hnRNP A1 dysfunction and tauopathy.

Normally localized in the nucleus, under conditions of cellular stress, hnRNP A1 can shuttle to the cytoplasm, where it participates in the formation of stress granules [19]. These granules allow for the temporary sequestration of nascent RNA transcripts during stress-induced translation arrest. Mutations associated with an elevated risk of proteinopathies are thought to enhance the cytoplasmic retention of hnRNP A1 and facilitate its separation into stress granules or other cytoplasmic condensates [20]. High protein concentrations within condensates may promote further transition of hnRNP A1 into insoluble amyloid-type aggregates–cross-beta fibrils [21]. Surface tension or similar factors may also be at play, considering that fibrillation typically begins at the interphase between condensates and the bulk solution [22,23].

**Figure 1:**
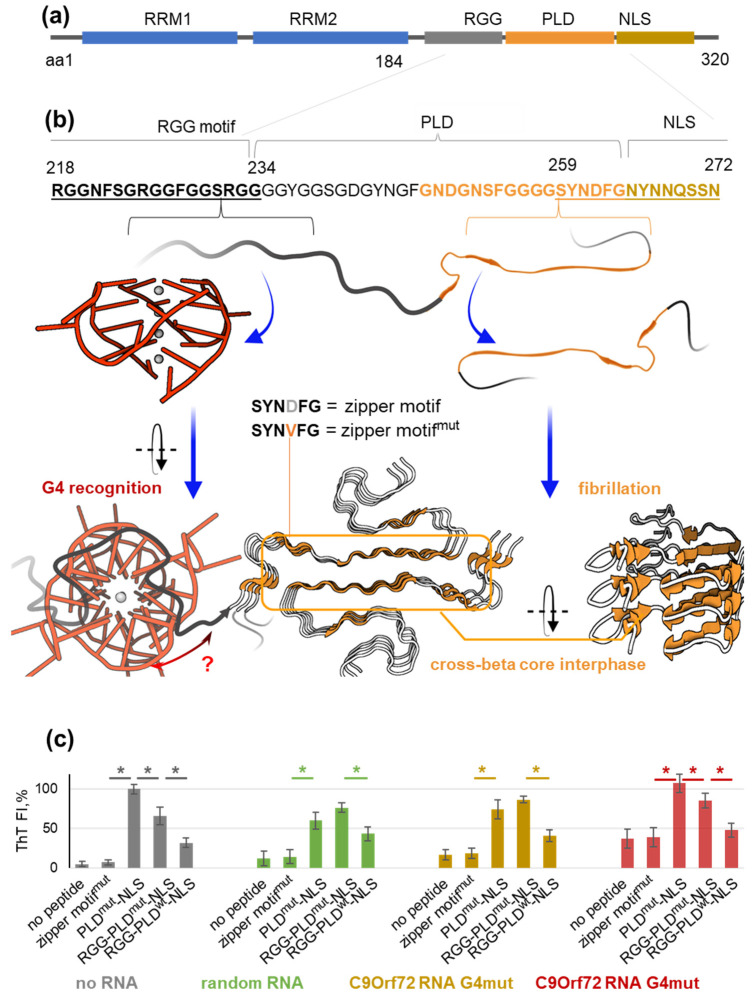
The domains and key motifs of hnRNP A1 are involved in G4 recognition and fibrillation. (**a**) The domain structure of hnRNP A1. (**b**) The schematic representation of hnRNP A1 fibrillation and binding to the C9Orf72 RNA G4. Blue, RNA recognition motifs of hnRNP A1 (RRM1 and RRM2); gray, Gly-rich disordered region of hnRNP A1; black, RGG motif within the Gly-rich region; orange, prion-like domain (PLD); yellow, nuclear localization signal (NLS); red, C9Orf72 RNA G4 (PDB ID: 8X0S). The orange box indicates the cross-beta core of hnRNP A1 (PDB ID: 7BX7). The blue arrows indicate potential interactions, and the black arrows indicate rotation. (**c**) ThT assays illustrating fibrillation of hnRNP A1 fragments in the presence/absence of random/G4 RNA. Conditions: 40 µM peptide, 13 µM ThT, 0.5 mM DTT, and 0.1 mg/mL random RNA or 20 µM G4 in 40 mM HEPES-KOH buffer (pH 7.4), supplemented with 150 mM KCl. Error bars indicate the SD of six measurements (two biological and three technical repeats). * *p* < 0.05 (two-tailed *t*-test).

The propensity of hnRNP A1 for LLPS is attributed to the transient interactions of its disordered LCD [21], which neighbors the N-terminal tandem RRMs and is followed by the C-terminal nuclear localization signal (NLS) M9 (Figure 1a). The LCD is composed of a glycine-rich region with several RGG repeats (the RGG motif) and an M9-flanking region enriched in non-charged polar amino acid residues. Such LCDs are commonly referred to as prion-like domains (PLD) because they bear compositional similarity to the yeast prion protein and often contain steric zipper motifs that can adopt beta-sheet conformations upon dimerization or intramolecular folding [24]. Initially unstable, the beta-sheets become energetically favorable if stacked on top of each other, which yields cross-beta fibrils.

A potential zipper motif of hnRNP A1, SYNDFG, was previously identified within the PLD [25]. The D/V substitution in this motif is prevalent in patients with FTD/ALS and has been shown to promote fibrillation of short zipper peptides in vitro. Subsequent studies of full-size hnRNP A1 fibrils by cryo-EM showed that both the zipper motif and M9 NLS are involved in the formation of the cross-beta core [26,27] (Figure 1b). This provides a partial explanation for the relationship between hnRNP A1 nucleoplasm-cytoplasmic shuttling and fibrillation propensity. Recognition of the NLS by nuclear import receptors (NIRs) is expected to stabilize its non-pathogenic conformation, as has been reported for other hnRNPs, such as TDP43 [28].

The chaperone-like activity of NIRs can be inhibited by proline-rich peptides, such as dipeptide repeats (PR)_n_ or (PG)_n_ generated from (G_4_C_2_)_n_ RNA via non-AUG translation [29]. The G_4_C_2_ repeats are located in the *C9Orf72* locus, and their expansion is associated with an increased risk of proteinopathies, most notably ALS/FTD [30]. Their contribution to pathology development may be multipath. In addition to competing with hnRNPs for NIRs [28], polydipeptides, especially R-rich ones, can interact with the LCDs of hnRNPs, including that of hnRNP A1 [31], and alter the assembly or dynamics of hnRNP-dependent condensates, including nuclear speckles and stress granules [32]. Furthermore, C9Orf72 RNA itself may be toxic [33]. It is implicated in the modulation of hnRNP-dependent LLPS [34] and can even form degradation-resistant wires [35], gel-like structures [36], or condensates in a protein-independent manner [37].

The unique properties of C9Orf72 RNA are typically attributed to its folding into G-quadruplex (G4)–planar arrangements of guanine tetrads [38]. For synthetic C9Orf72 oligonucleotides, only intermolecular (tetrameric) structures have been reported thus far [39,40]. However, the intramolecular folding of RNA transcripts cannot be excluded. It is also possible that the RNA G4s are conformationally polymorphic, similar to their DNA counterparts [41], and interspersed with unfolded RNA regions. In any case, the G4s are present in C9Orf72 RNA, and their terminal tetrads must be accessible for pi-pi and cation-pi interactions, which are prevalent in biomolecular condensates [42].

C9Orf72 RNA has been shown to interact with several hnRNPs, including hnRNP H and FUS, in tubes and to affect their phase transitions in cells [35,43]. However, its direct interaction with hnRNP A1 has not yet been tested. We aimed to investigate the possible mutual effects of hnRNP A1 and C9Orf72 G4 in vitro (Figure 1b). In particular, we examined the impact of the G4 on the SRSF-dependent LLPS of wild-type hnRNPA1, which mimics speckle assembly in the nucleus, and on SRSF-independent fibrillation of mutant hnRNP A1 fragments, which mimics the assembly of cytoplasmic cross-beta structures. As hnRNPs tend to exhibit G4 helicase-like activity [44,45], we also tested the effects of hnRNP A1 on C9Orf72 G4 folding.

## 2. Results and Discussion

### 2.1. The RGG Motif Restricts the Spontaneous Fibrillation of the PLD-NLS Fragment of HnRNP A1

Our first goal was to elucidate the potential impact of C9Orf72 RNA on the spontaneous fibrillation of hnRNP A1, which is expected in the case of cross-beta-stabilizing PLD mutations. We considered the mutant hexapeptide sequence SYNVFG (zipper motif^mut^; aa 259–264) [46] and its homologue with flanking PLD and NLS regions (PLD^mut^-NLS; aa 234–272) from the cross-beta interface (Figure 1b) [27] as minimal fibrillation-prone hnRNP A1 fragments. Fibrillation was verified using the cross-beta core-binding fluorogenic probe thioflavin T (ThT) [22] at room temperature, according to a previously described protocol [25] (Figure 1c). In line with the cryo-EM-based evidence for the importance of zipper flanks [27], the hexapeptide zipper motif^mut^ showed no signs of fibrillation, whereas PLD^mut^-NLS induced a pronounced ThT light-up rapidly after mixing, and the fluorescence signal remained nearly constant for at least 3 h (Figure S1).

Subsequently, we tested the extended peptide RGG-PLD^mut^-NLS (aa 218–272; Table 1) at room temperature (Figure 1c) and at 37 °C (Figure S2) to verify the impact of the RGG motif. Although this motif is not directly involved in the formation of the cross-beta core, we hypothesized that it may influence the LLPS-fibrillation equilibrium, given its LLPS-prone patterned primary structure and irregular secondary structure. The ThT signal in the RGG-PLD^mut^-NLS sample was indeed reduced by 30 ± 10% (*p* < 0.05, *t*-test) relative to the PLD^mut^-NLS (Figure 1c and Figure S2, gray bars), suggesting that RGG serves to partly counterbalance the propensity of PLD-NLS for fibrillation. This observation is consistent with the effects of other disordered biopolymers, such as poly-ADP-ribose (PAR). For instance, stress-induced PARylation of hnRNP A1 has been shown to facilitate its maintenance in condensates (stress granules) rather than in solid aggregates [47]. Presumably, disordered mixed-sequence RNA from yeast (hereafter referred to as random RNA) also inhibited the fibrillation of PLD^mut^-NLS (Figure 1c and Figure S2, green bars). However, it did not affect RGG-PLD^mut^-NLS or its wild-type analog RGG-PLD^wt^-NLS. 

Notably, the fibrillation of the latter, both with and without random RNA, was approximately two times lower than that of RGG-PLD^mut^-NLS, but still significant. We conclude that even non-mutant hnRNP A1 can undergo minor spontaneous fibrillation, and that the protective effect of RGG is rather limited.

The hnRNP A1 peptides were then screened for sensitivity to RNA G4s (Table 1). In addition to the wild-type C9Orf72 RNA fragment comprising four G4 tracts (C9Orf72 RNA G4wt) capable of forming extra-stable structures with at least four G-tetrads [40,41], we analyzed a homologous sequence comprising four G3 tracts (C9Orf72 RNA G4mut) and presumed to adopt a less stable structure with fewer tetrads. Both RNA sequences were tested for ThT light-up to verify their folding (Figure 1c, yellow and red bars). Most G4s induce noticeable ThT light-up effects, comparable to those observed in fibrils, and the intensity of the fluorescent signal is proportional to the G4 fraction in the first approximation [48]. The ThT signal of the peptide-free C9Orf72 RNA G4wt sample was markedly higher than those of the C9Orf72 RNA G4mut and random RNA samples (*p* < 0.05, *t*-test), supporting the stability of G4wt. A similar trend was observed for the G4wt/G4mut/random RNA mixtures with the zipper motif^mut^ or PLD^mut^-NLS. In contrast, the mixtures of RGG-PLD^mut^-NLS or RGG-PLD^wt^-NLS with the G4s provided no additional light-up compared to the G4-free samples. In other words, the signal was non-additive and lower than expected. These observations can be explained by inhibition of fibrillation by the G4 in an RGG-dependent manner, RGG-dependent unwinding of the G4, or both. To clarify this issue, we next investigated the samples by atomic force microscopy (AFM).

### 2.2. The C9Orf72 RNA G4 Modulates Fibrillation of RGG-PLD-NLS, Favoring Smaller Aggregates

**Figure 2:**
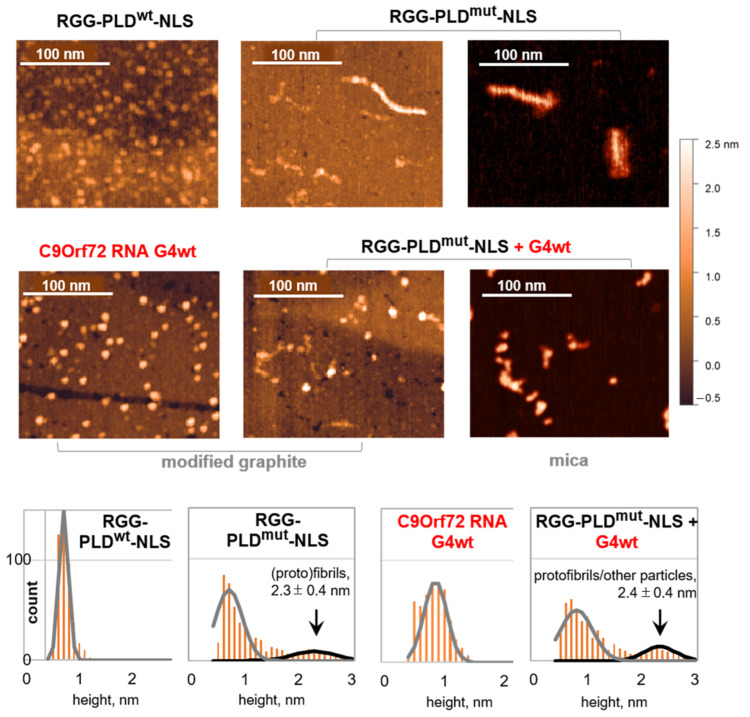
The impact of the RNA G4 on the fibrillation of hnRNP A1 (RGG)-PLD^mut^-NLS. AFM assays. Top: representative AFM images of the samples from the ThT assays on a modified graphite support or mica. Bottom: summary of the statistical analysis of particle heights in the AFM images obtained on a modified graphite support (500 particles for each sample type). All ThT assays and AFM scans were performed after a 3 h incubation of the peptide/RNA solutions or their mixtures at room temperature.

A comparative analysis of RGG-PLD^mut^-NLS fibrillation in the presence and absence of C9Orf72 RNA G4wt was performed using AFM on a modified graphite surface, which is suitable for both proteins and RNA [49,50]. The representative AFM images are shown in Figure 2, and the additional large-field images are provided in Figure S3. Free C9Orf72 RNA G4wt was visualized as small granules with a diameter of 0.9 ± 0.2 nm, along with occasional larger granules up to 1.5 nm. These morphologies are consistent with monomers and dimers or stacked monomers, but not multimolecular G-wires [37].

The G4-free RGG-PLD^mut^-NLS sample contained small irregularly shaped particles/strings (monomeric peptides), granules (protofibrils, amorphous aggregates, or condensate seeds), and fibrils of up to several hundred nanometers in length. The apparent height of these fibrils (2.3 ± 0.4 nm) was lower than that of full-length hnRNP A1 fibrils (7 nm, according to cryo-EM data [26], or 5 nm, according to AFM data [22]), but close to that of hnRNP A2 PLD fibrils (2.9 nm) [51]. It was also markedly higher than that of monomeric peptides (<1 nm), which dominated in the control RGG-PLD^wt^-NLS sample.

In the mixture of RGG-PLD^mut^-NLS and G4wt, the predominant species were individual granules (G4s) and small irregularly shaped objects (monomeric peptides). However, granules with protruding “tails” (presumably complexes) and short particles of 2.4 ± 0.4 nm height (protofibrils, condensate seeds, or peptide-G4 complexes) were also present. The number of large (>2 nm) particles increased by approximately 30% in comparison to the free RGG-PLD^mut^-NLS sample, yet no lengthy fibrils were observed. To eliminate any potential artifacts associated with the distinct sorption of monomers, oligomers, complexes, and fibrils on the graphite surface, we conducted a second analysis using a different support (mica) and obtained similar results (Figure 2 and Figure S3). The G4 shifted the dominant type of large particles from fibrils to granules and irregularly shaped particles, irrespective of the support used.

We conclude that the G4 restricts the fibrillation of RGG-PLD^mut^-NLS and thus exhibits a holdase-like activity, probably due to the partial shielding of the cross-beta interface upon interaction with RGG. Alternatively, the G4 could trap RGG-PLD^mut^-NLS in a kinetically favored conformation. Similar effects have been previously reported for other parallel-stranded and hybrid-topology G4s [52]. To gain some insight into the potential conformational changes upon the interaction between RGG-PLD^mut^-NLS and the G4, we proceeded to conduct circular dichroism (CD) spectroscopy studies.

### 2.3. RGG-PLD-NLS Unfolds C9Orf72 RNA G4 and Its Mutant at Micromolar Concentrations

**Figure 3:**
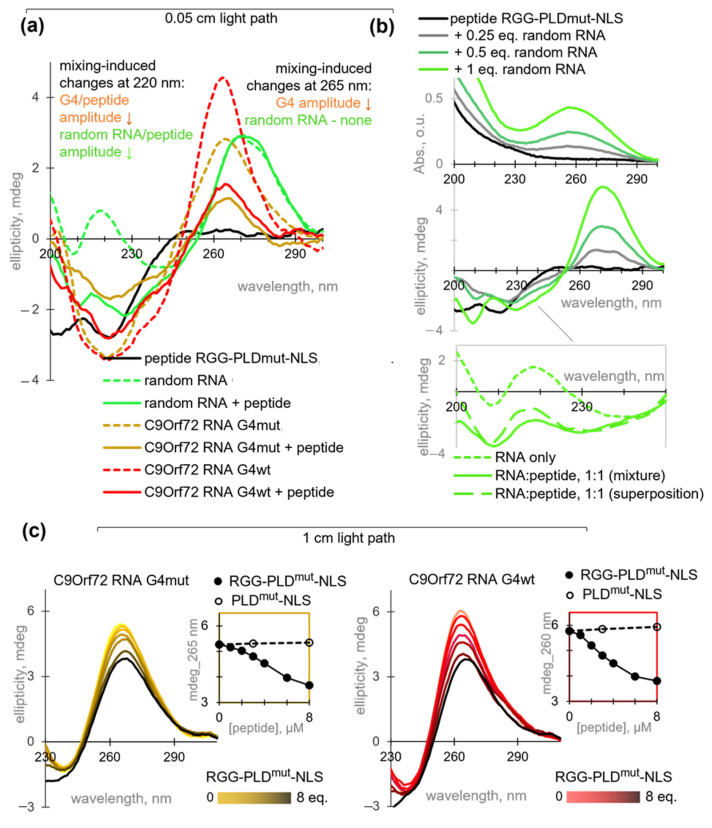
The impact of RGG-PLD^mut^-NLS on the RNA secondary structure and vice versa. (**a**) CD spectra of RGG-PLD^mut^-NLS (40 µM), random RNA (0.1 mg/mL), G4wt (10 µM), G4mut (10 µM), and their mixtures in 4 mM HEPES-KOH buffer (pH 7.4) supplemented with 15 mM KCl (0.1 × HEPES buffer). (**b**) Ellipticity changes upon RGG-PLD^mut^-NLS titration with random RNA. Conditions: 0.2 mg/mL peptide and 0–0.2 mg/mL RNA in 0.1 × HEPES buffer. Top: UV absorption spectra; middle: CD spectra; bottom: comparison of the experimental (mixture) and theoretical (superposition) spectra in the far-UV region. (**c**) Ellipticity changes upon G4mut (**left**) or G4wt (**right**) titration with the peptides. Conditions: 1 µM G4 and 0–8 µM peptide in 0.1 × HEPES buffer. All spectra were registered at room temperature in 0.05 cm (**a**,**b**) or 1 cm (**c**) cuvettes (the average of three measurements is shown).

First, we performed CD measurements at medium micromolar peptide/RNA concentrations, which were necessary for peptide fibrillation. The spectrum of RGG-PLD^mut^-NLS (Figure 3a) contained a characteristic negative band at 220 nm, indicative of beta-sheets, and an additional negative band at 200 nm, ascribed to the disordered part of the peptide [53]. Deconvolution of the spectrum yielded dominant contributions of antiparallel beta-sheets and random coils (34 ± 3% each) with 20 ± 1% beta-turns and minor contributions of parallel beta-sheets and alpha helices. These features are consistent with the cryo-EM-based model of the fibril core flanked by a disordered RGG motif (Figure 1). The spectrum of C9Orf72 RNA G4wt was consistent with a parallel-stranded G4 folding, with a large positive band at 265 nm and a smaller negative band at 220 nm. The control sample, C9Orf72 RNA G4mut, exhibited a comparable spectrum, with the exception of a diminished amplitude at 265 nm, indicative of fewer stacked G residues [41]. In contrast, the random RNA sample exhibited a positive CD band at 275 nm, which is characteristic of ssRNA and dsRNA [54,55]. 

The contribution of the peptide to the near-UV CD amplitude at the RNA secondary structure-specific maxima (265–275 nm) was negligible, thereby allowing direct monitoring of peptide-induced RNA rearrangements. In contrast, the amplitudes of the RNA signatures in the far-UV range were considerable. Consequently, it was impossible to monitor RNA-induced peptide rearrangement directly. We could only compare the spectra of the mixtures with the superposition of the individual components. In the near-UV region, the spectrum of the peptide mixture with random RNA (2:1 mass ratio) was similar to that of pure RNA, indicating no peptide-induced rearrangements or random RNA (Figure 3a). In the far-UV region, the negative band increased with increasing concentrations of random RNA, but the signal was non-additive. Specifically, the 1:1 peptide: RNA mixture exhibited a higher amplitude of the negative band near 200 nm than that expected for the superposition of pure peptide/RNA signatures, indicating a slight RNA-induced shift toward a random coil (Figure 3b). In contrast, the spectra of the peptide mixtures with G4wt or G4mut were clearly distinct from the superpositions in both the far-UV and the near-UV regions. The negative signal at 220 nm was non-additive and lower than expected, indicating the disruption of beta-sheets, G4s, or both. The positive signal at 265 nm was also lower than expected, supporting G4 unwinding (Figure 3a).

Next, to additionally verify the G4 unwinding, we focused on the near-UV range and studied the G4 samples at a low concentration (1 µM), which is more relevant at the cellular level and favors intramolecular folding over oligomerization (Figure 3c). The optical path was increased to maintain the signal-to-noise ratio. The G4mut sample exhibited nearly identical molar ellipticity values at 1 µM and 10 µM concentrations ([θ]^G4mut^_1 µM_ = 530 deg∙cm^2^/dmol and [θ]^G4mut^_10 µM_ = 560 deg∙cm^2^/dmol). In contrast, the G4wt sample exhibited a lower molar ellipticity at a concentration of 1 µM than at 10 µM ([θ]G4^wt^_1 µM_ = 620 deg∙cm^2^/dmol versus [θ]G4^wt^_10 µM_ = 890 deg∙cm^2^/dmol), although still higher than that of the G4mut sample. These findings suggest that G4wt undergoes oligomerization at medium micromolar concentrations, while G4mut remains unaltered.

Titration of the diluted (1 µM) G4 samples with RGG-PLD^mut^-NLS resulted in a gradual decrease in the 265 nm CD band, accompanied by a minor bathochromic shift that became noticeable at 6–8 eq. peptide. These observations confirm the partial unwinding of the G4 structure. It is noteworthy that the peptide acted on both intramolecular and intermolecular G4s, yet their unwinding was incomplete and required a relatively high concentration of RGG-PLD^mut^-NLS (Figure 3c). Importantly, PLD^mut^-NLS failed to unwind the G4s (Figure S6), underscoring the role of RGG. Previous studies have demonstrated that full-length hnRNP A1 can bind and unwind other RNA and DNA G4s, such as those present in telomeres [56,57] and the KRAS promoter [58]. This helicase-like activity is typically ascribed to the RGG motif [59], which is known to engage in cation-pi interactions with the G4 aromatic planes and electrostatic interactions with the DNA/RNA backbone. It is possible that aromatic residues located between the RGG repeats (specifically, F residues in the case of hnRNP A1) are involved in this process by forming stacking contacts with guanine residues. Importantly, the RGG-neighboring RRMs have also been implicated in G4 binding [60]. However, our data argue against the necessary involvement of RRMs in the case of hnRNP A1.

### 2.4. C9Orf72 RNA G4 Interferes with SR-Dependent Phase Separation of HnRNP A1 into Biocondensates

Following an investigation into the sensitivity of C9Orf72 RNA G4 to RGG and its impact on the pathological phase transitions of the RGG-neighboring PLD, we proceeded to test the impact of the G4 on the PLD-driven transition that is critical for its normal function, namely the co-separation with SRSFs and pre-mRNA/ncRNA in nuclear speckles. In the model speckles, we used a combination of random RNA and an SR-rich fragment of SRSF1 (SRSF^fr^, Table 1), which exhibits high homology with the SR fragments of other SRSF family members. To visualize the model speckles by fluorescence microscopy, the admixtures of hnRNP A1 labeled with RED-tris-NTA (for imaging in a red channel) and FITC-labeled SRSF^fr^ (for imaging in a green channel) were added to the unlabeled proteins.

**Figure 4:**
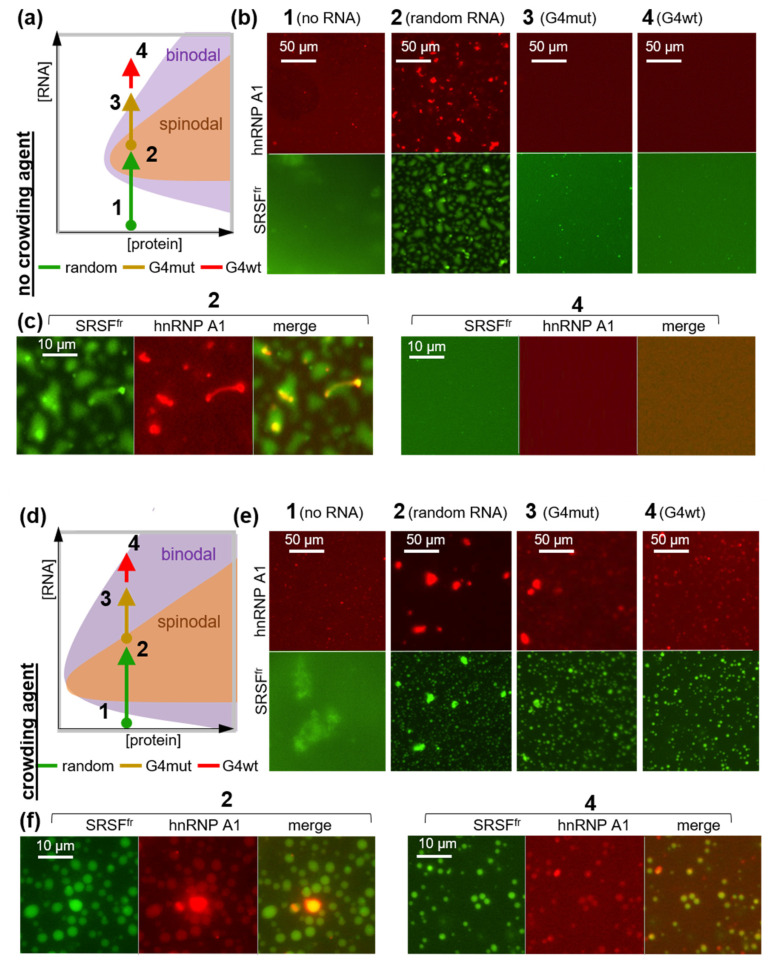
The impact of the G4s on the co-separation of hnRNP A1 and SRSF^fr^. (**a**) A generic phase diagram of an RNA-protein mixture in non-crowded media. The arrows between points 1 and 4 summarize the experimental setup and the observed RNA-induced transitions. (**b**) Fluorescence microscopy images of the hnRNP A1/SRSF^fr^ mixture with/without RNA. Conditions: 6 µM hnRNP A1 (5% RED-labeled) and 1 mg/mL SRSF^fr^ (5% FITC-labeled) in the absence of RNA (1), in the presence of 3 mg/mL random RNA (2), in the presence of 3 mg/mL random RNA and 6 µM G4mut (3), or in the presence of 3 mg/mL random RNA and 6 µM G4wt (4). All images were obtained after 30 min of incubation of the mixtures at room temperature in 40 mM HEPES-KOH buffer (pH 7) supplemented with 150 mM KCl. (**c**) Enlarged images of samples 2 (random RNA only) and 4 (random RNA with G4wt) in the far-red (hnRNP A1) and green (SRSF^fr^) channels and their superposition. (**d**) A generic phase diagram of an RNA-protein mixture in a crowded media. (**e**) Fluorescence microscopy images of hnRNP A1/SRSF^fr^ mixture with/without RNA in the presence of a crowding agent (PEG-400). The conditions are similar to those in (**b**), except that 10% PEG-400 was added to all samples. (**f**) Enlarged images of samples (2) and (4) in the presence of PEG obtained from the red and green channels and their superposition.

Model speckles were assembled using 6 µM hnRNP A1, which was well below the concentration required for fibrillation. In contrast, SRSF^fr^ was used in large excess (1 mg/mL), given the presumed prevalence of this protein in nuclear speckles. In the absence of RNA, only a few small irregularly shaped aggregates were observed in the SRSF^fr^ and hnRNP A1 samples, and their mixture showed no signs of co-separation (point 1 in Figure 4a,b). The addition of random RNA at concentrations up to 1 mg/mL had no significant impact; however, its excess induced the formation of condensates/aggregates of several µm (point 2 in Figure 4a–c). Interestingly, the hnRNP A1 condensates/aggregates were located within those of SRSF^fr^, which exhibited a classic spinodal separation (Figure 4c). This finding aligns with the prevailing model of nuclear speckles with hnRNP A1 inclusions in SRSF-scaffolded condensates [9]. However, it does not fully align with the multilayer model, which posits that hnRNP A1 coats the SRSF core [61]. The addition of G4mut (point 3 in Figure 4a,b) or G4wt (point 4 in Figure 4a–c) to a final concentration of 6 µM (1 eq. relative to hnRNP A1) to the random RNA-SRSF^fr^-hnRNP A1 mixture resulted in inhibition of phase separation. The G4mut-containing sample displayed residual submicrometer condensates/aggregates that were visible in the FITC-SRSF^fr^ channel, whereas the G4wt-containing sample exhibited a diffuse distribution of both SRSF^fr^ and hnRNP A1 (Figure 4c). Given the excess SRSF^fr^, this effect was attributed to G4 interactions with hnRNP A1. When engaged in contact with the G4, RGG-PLD must be inaccessible for transient interactions with random RNA and SRSF^fr^. It is important to note that the observed inhibitory effect of the G4s on the phase separation may be model-specific. The principal advantage of our in vitro model is the straightforward interpretation of the observed transitions (Figure 4a) due to the minimal number of components involved. However, its relevance to native nuclear speckles is limited. The typical morphology of native nuclear speckles is intermediate between spinodal and bimodal [61]. 

To enhance the precision of our model and partially account for the non-essential proteins that may induce molecular crowding [62], we added polyethylene glycol (PEG-400) as a crowding agent and repeated all the experiments (Figure 4d–f). In the first approximation, molecular crowding is analogous to increasing the concentrations of both protein and RNA components, as illustrated by a shift in the generic phase diagram along both axes (Figure 4d). The spontaneous association of proteins into amorphous aggregates (SRSF^fr^) or submicrometer aggregates of indistinguishable shape (hnRNP A1) was enhanced in the presence of PEG. However, in the absence of RNA, there were no indications of hnRNP A1-SRSF^fr^ co-separation (point 1 in Figure 4d,e), as in the PEG-free samples. Random RNA (point 2 in Figure 4d–f) induced the formation of multiple micrometer-sized droplets, which gradually coalesced into larger (up to 10 µm) structures (Figure 4f). Thus, PEG induced a transition from spinodal to binodal phase separation in the presence of random RNA (Figure 4a,d and Figure S4). The subsequent addition of G4mut resulted in a decreased number of large droplets (point 3 in Figure 4d,e), while the addition of G4wt (point 4 in Figure 4d–f) inhibited their formation altogether. Instead, multiple submicrometer droplets, which nevertheless retained the hnRNP A1-SRSF^fr^ colocalization, were observed (Figure 4f and Figure S5). Importantly, despite the maintenance of small droplets, the partitioning coefficient (P) of hnRNP A1 was reduced by 60 ± 5% in the presence of the G4s (P_no G4_ = 4.2 ± 0.1 versus P_G4wt/mut_ = 1.6 ± 0.2). We concluded that the C9Orf72 RNA G4wt and, to a lesser extent, G4mut inhibited the co-separation of hnRNP A1 and SRSF^fr^.

**Figure 5:**
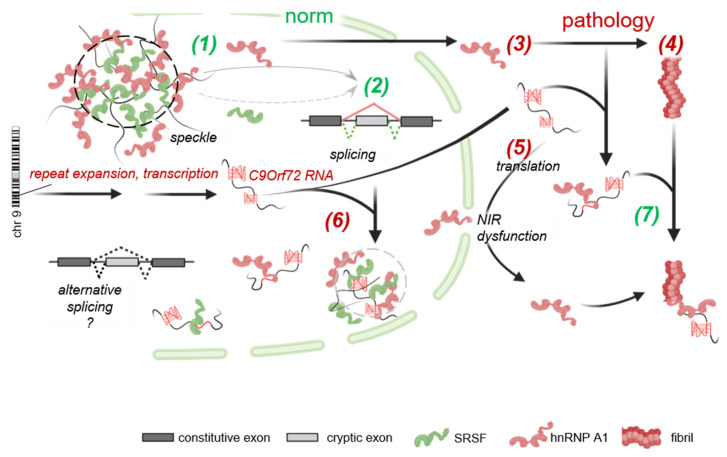
Hypothetical intracellular transitions of hnRNP A1 in the presence of C9Orf72 RNA G4. Normally, hnRNP A1 co-separates with SRSFs and pre-mRNA/ncRNA in nuclear speckles (1), which ensures its homeostasis in the nucleoplasm and its controlled contribution to the splicing of cryptic exons (2). Under stress conditions, the relocation of hnRNP A1 to the cytoplasm (3) opens ways for its fibrillation (4). The maintenance of hnRNA 1 in the cytoplasm is possible upon the accumulation of C9Orf72 RNA, which is prone to non-AUG translation into NIR-inhibiting polydipeptides (5). At the same time, C9Orf72 RNA is likely to disrupt the co-separation of hnRNP A1 with SRSFs in the nucleoplasm (6), suggesting an increased risk of splicing deregulation (loss-of-function toxicity). In the cytoplasm, C9Orf72 RNA is likely to form irregular aggregates/condensates, but not lengthy fibrils, with hnRNP A1 (7), suggesting a decreased risk of fibrillation (gain-of-function toxicity).

The interpretation of these in vitro results with respect to the intracellular roles of hnRNP A1 under normal and pathological conditions is presented in Figure 5. The accumulation of C9Orf72 RNA and, to a lesser extent, other G4s is expected to disrupt nuclear speckles (Figure 4), altering the nucleoplasmic hnRNP A1:SRSF ratio and the spliceosome assembly at weak splicing sites. The enhanced release of hnRNP A1 from speckles may result in its accumulation in the cytoplasm. The products of C9Orf72 RNA non-AUG translation are reportedly capable of exacerbating this effect by preventing hnRNP A1 binding to NIRs [29], thus increasing the likelihood of pathological fibrillation in the cytoplasm. However, as demonstrated by our in vitro assays (Figure 2), RNA *per se* is unlikely to induce hnRNP A1 fibrillation.

## 3. Materials and Methods

### 3.1. Peptides, Proteins, Oligonucleotides, and Reagents

Oligonucleotides C9Orf72 RNA G4wt and C9Orf72 G4mut (Table 1) were purchased from Evrogen (Moscow, Russia). Torula yeast-derived mixed-sequence RNA (random RNA) was purchased from HiMedia Laboratories (Thane, India). His-tagged full-length recombinant human hnRNP A1 was purchased from AMSBIO (Abingdon, UK). For fluorescence microscopy imaging, the protein was labeled with an RED dye residue (NanoTemper Technologies, Munich, Germany), which emits light in the far-red region upon excitation at 647 nm, using the RED-tris-NTA kit (NanoTemper Technologies, Munich, Germany) following the manufacturer’s instructions. 

Peptides zipper motif^mut^, PLD^mut^-NLS, RGG-PLD^mut^-NLS, RGG-PLD^wt^-NLS, and SRSF^fr^ (hnRNP A1 fragments, Table 1) were synthesized on a Liberty Blue Synthesizer (CEM corporation, Matthews, USA) following the Fmoc strategy and standard protocols for the solid-phase peptide synthesis with microwave treatment of the reaction mixture. Fmoc-Gink-Amide PEG resin (Iris Biotech, Marktredwitz, Germany) was used as the solid-phase support. Activation of Fmoc-amino acids and coupling were performed using ethyl (hydroxyimino)cyanoacetate (0.5 M) in the presence of N,N’-diisopropylcarbodiimide (0.25 M). Fmoc Protecting groups were removed by treatment with 20% piperidine in DMF. The cleavage step was performed with trifluoroacetic acid in dichloromethane using triisopropylsilane and 2,2′-(ethylenedioxy)diethanethiol as scavengers.

The peptide was purified by RP-HPLC (purity > 90%) on an Acta Pure chromatography system (GE Healthcare Life Sciences, Chicago, USA) using Zorbax SB-C18 columns, 9.4 × 250 mm, 5µ (Agilent, Santa Clara, CA, USA), and the purity was verified using the LCMC-2020 system (Shimadzu, Tokyo, Japan). The peptides were lyophilized and stored at −20 °C. Thioflavin T (ThT) for fibrillation assays was purchased from Sigma-Aldrich (Darmstadt, Germany). For fluorescence microscopy imaging, peptide SRSF^fr^ was labeled with fluorescein isothiocyanate (FITC). The peptide was dissolved in 0.1 M sodium-carbonate buffer (pH 9.0) to a concentration of 2 mg/mL, and FITC solution in dry DMSO was added dropwise to a final FITC concentration of 0.05 mg/mL. The mixture was incubated overnight at 4 °C and the reaction was quenched by the addition of 50 mM ammonium chloride.

### 3.2. Fibril Assembly and ThT Assays

To obtain fibrils, fresh stock solutions of the peptides (hnRNP A1 fragments) in MilliQ water were mixed with the stock HEPES-KOH buffer (pH 7.4) to a final peptide concentration of 40 µM. The final composition of the working buffer was 40 mM HEPES-KOH, 150 mM NaCl, and 0.5 mM DTT. (For assays with RNA, the buffer was prepared in DEPC-treated water.) Thioflavin T (ThT) (Sigma-Aldrich, Darmstadt, Germany) was added to a final concentration of 13 µM. The mixtures were stored in the dark for at least 3 h. For kinetics analysis, the ThT signal was measured every 15–60 min. ThT fluorescence emission was measured at 490 nm upon excitation at 450 nm using an Infinite 200 PRO plate reader (Tecan, Männedorf, Switzerland).

For the evaluation of RNA effects on fibrillation, random RNA was added to the peptide samples to a final concentration of 0.1 mg/mL prior to ThT addition. To verify G4 folding, ThT was added to pre-annealed solutions of C9Orf72 RNA G4wt or G4mut (20 µM) in 40 mM HEPES-KOH buffer (pH 7.4) supplemented with 150 mM KCl to a final ThT concentration of 13 µM. Rapid annealing (heating to 90 °C for 5 min, followed by snap-cooling on ice) was used to facilitate intramolecular G4 folding. All experiments were performed at least twice (on different days, different peptide/RNA aliquots) with 3 technical replicates in each case.

### 3.3. Atomic Force Microscopy (AFM)

For verification of the G4 impact on fibrillation, samples of the peptide (40 µM) and/or G4 (20 µM) in the HEPES/KOH working buffer were centrifuged (10,000× *g*, 20 min) and applied onto mica or the freshly cleaved graphite surface rendered hydrophilic by pretreatment with an amphiphilic reagent (CH_2_)_n_(NCH_2_CO)_m_-NH_2_. AFM images were obtained in air using homemade super-sharp cantilevers (64) and a multimode AFM instrument with an NTEGRA Prima controller (NT-MDT, Russia) in the tapping mode with a typical scan rate of 1 Hz and a typical free amplitude of several nanometers. The AFM data were filtered and analyzed using the FemtoScan Online software, version 2.3.239 (5.2) (ATC, Moscow, Russia), and then flattened using standard algorithms (subtraction of the quadric surface and averaging by lines). The apparent heights of the fibrils and other particles were calculated using Gwyddion software, version 2.58.

### 3.4. Circular Dichroism (CD) Spectroscopy

CD spectra of RNA, peptides, and their mixtures were registered using a Chrisacan spectrofluorometer (Applied Photophysics, Leatherhead, UK) at 20 °C in quartz cuvettes of 0.05 nm light path (for the analysis of the far-UV region) or 1 cm path (for the near-UV region). For optimal signal-to-noise ratio in the far-UV region, the peptide/RNA samples were prepared in low-ionic strength buffer (4 mM HEPES-KOH, pH 7.4, and 15 mM KCl). The far-UV region was analyzed for the pure peptide RGG-PLD^mut^-NLS (40 µM = 0.2 mg/mL) random RNA (0.1 mg/mL), pre-annealed C9Orf72 RNA G4mut/wt (10 µM), and their mixtures after 1 h or incubation. The spectrum of the peptide was deconvoluted using CDNN software, version 2.1.0.223. The unfolding of the G4s was monitored in the near-UV region upon titration of the 1 µM sample with increasing concentrations of the peptide (up to 8 µM). The measurements were performed in triplicate, and the spectra were averaged after background subtraction.

### 3.5. Biocondensate Assembly and Fluorescence Microscopy Imaging

For biocondensate assembly, full-length hnRNP A1 (95% unlabeled and 5% labeled with RED-tris-NTA) was mixed with unlabeled SRSF^fr^ (95% unlabeled and 5% FITC-labeled) in 40 µM HEPES-KOH buffer (pH 7.0), supplemented with 150 mM NaCl and 0.5 mM DTT. Then, RNA was added to a final concentration of 3 mg/mL. The final protein concentrations were 6 µM (hnRNP A1) and 1 mg/mL (SRSF), and 10% PEG-400 was added to mimic molecular crowding. To assess the impact of the G4s on biocondensate formation, C9Orf72 RNA G4wt or G4mut was added to a final concentration of 6 µM, and the samples were incubated for 30 min at room temperature prior to imaging. The condensates were visualized using an Eclipse Ti2 microscope (Nikon, Japan) upon RED excitation in the 647 nm band with a cut-on wavelength of 660 nm or FITC excitation in the 488 nm band with a cut-on wavelength of 520 nm. The partitioning coefficient was calculated using ImageJ (Fiji) software, version 1.54d. The experiments were performed in duplicate.

## 4. Conclusions

The relationship between hnRNP A1 and C9Orf72 RNA G4 was investigated in vitro. The hypothesis that the G4 affects phase transitions of hnRNP A1, namely its co-separation with SRSFs and fibrillation, was based on the proximity of the G4 binding motif (RGG) to LLPS/fibrillation-prone PLD-NLS. This assumption was tested using the full-length wild-type protein and its fragments (synthetic peptides). The mutation-dependent fibrillation of the peptides was confirmed by ThT assays, which also revealed a moderate inhibitory effect of RGG on fibrillation.

The C9Orf72 RNA G4 turned out to restrict fibril growth, as was evident from the AFM analysis of the RGG-PLD^mut^-NLS peptide with the fibrillation-promoting mutation in the zipper motif of its PLD. This holdase-like activity of the G4 was attributed to the partial shielding of PLD upon G4 interaction with RGG. The interaction also induced partial unwinding of the G4 and reduced its propensity for oligomerization. This helicase-like activity of RGG-PLD^mut^-NLS was evidenced by CD spectroscopy data. Collectively, these findings argue against the synergistic gain-of-function toxicity of hnRNP A1 and C9Orf72 RNA on C9Or72 repeat expansion and hnRNP A1 mutation.

The impact of the G4 on the non-mutant full-length protein was also consistent with the engagement of RGG-PLD. The G4 facilitated the release of hnRNP A1 from the speckle-mimicking condensates with the SR-rich peptide and RNA, as evidenced by fluorescence microscopy data. The increased release of hnRNP A1 from speckles is expected to prevent its function in the fine-tuning of alternative splicing. Therefore, our in vitro data support the G4-dependent splicing-related loss-of-function toxicity upon *C9Or72* repeat expansion.

## Figures and Tables

**Table 1:** Sequences of the peptides and oligonucleotides used in this study.

Code ^1^	Sequence	Position (Protein)
zipper motif^mut^	SYNVFG	259–264 (hnRNP A1)
PLD^mut^-NLS	GGGYGGSGDGYNGFGNDGSNFGGGGSYNVFGN YNNQSSN	234–272 (hnRNP A1)
RGG-PLD^mut^-NLS	RGGNFSGRGGFGGSRGGGGYGGSGDGYNGFGN DGSNFGGGGSYNVFGNYNNQSSN	218–272 (hnRNP A1)
RGG-PLD^wt^-NLS	RGGNFSGRGGFGGSRGGGGYGGSGDGYNGFGN DGSNFGGGGSYNDFGNYNNQSSN	218–272 (hnRNP A1)
SRSF^fr^	PRSPSYGRSRSRSRSRSRSRSRSNSRSRSYSP	197–228 (SRSF1)
C9Orf72 RNA G4wt	GGGGCCGGGGCCGGGGCCGGGG	N/A
C9Orf72 RNA G4mut	GGGCCGGGCCGGGCCGGG	N/A

^1^ Upper indexes in peptide codes: mut—the peptide contains the D262V mutation; wt—wild type (no mutation); fr—the fragment of the full length SRSF.

## Data Availability

The original contributions presented in the study are included in the article/supplementary material, further inquiries can be directed to the corresponding authors.

## Supplementary Materials

The following supporting information can be downloaded at https://www.mdpi.com/article/10.3390/molecules29184369/s1, Figure S1: Time-dependence of the fibrillation of mutant hnRNP A1 fragments monitored by ThT assays; Figure S2: ThT assays after incubation of hnRNP A1 (RGG)-PLDmut-NLS with and without RNA at a room temperature (light bars) and 37 °C (dark bars); Figure S3: The impact of C9Orf72 RNA G4 on the fibrillation of the mutant hnRNP A1 fragment: large-field atomic force microscopy (AFM) images; Figure S4: The impact of the crowding agent on the co-separation of hnRNP A1 and SRSF^fr^ in the presence of random RNA: large-field fluorescence microscopy images; Figure S5: The impact of the G4 on the co-separation of hnRNP A1 and SRSF^fr^ in the presence of random RNA in the crowded environment: large-field fluorescence microscopy images; Figure S6: Ellipticity changes upon G4mut (left) or G4wt (right) titration with the peptide PLD^mut^-NLS.
