# Supplementary material for: Cross-Effects in Folding and Phase Transitions of hnRNP A1 and C9Orf72 RNA G4 In Vitro
Source: Molecules. 2024 Sep 14;29(18):4369. doi: 10.3390/molecules29184369 (PMC11434081; doi:10.3390/molecules29184369)
Supplement: Supplementary file 1 [file molecules-29-04369-s001.zip › molecules-3183543-supplementary.pdf]

SUPPORTING INFORMATION

Cross-effects in folding and phase transitions of hnRNP A1 and C9Orf72 RNA G4 *in vitro*

Vedekhina T.S.*, Svetlova J.I., Pavlova Iu.I., Alieva S.A., Barinov N.A., Malakhova E.I., Rubtsov P.V., Shtork A.S., Klinov D.V., Varizhuk A.M.*

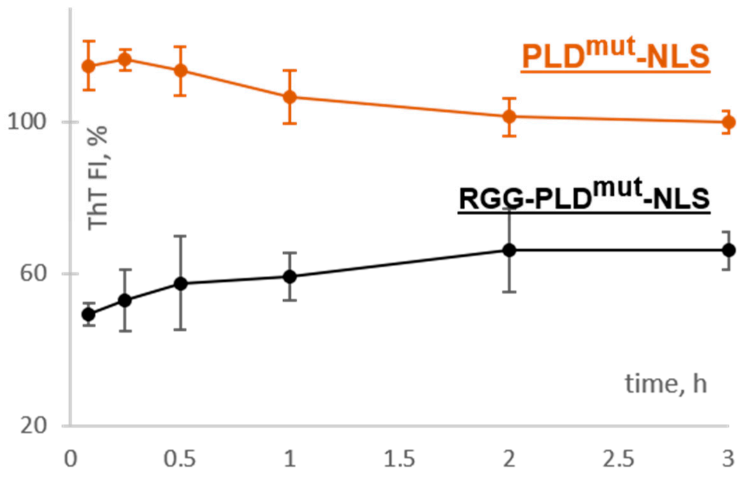

Figure S1. Time-dependence of the fibrillation of mutant hnRNP A1 fragments monitored by ThT assays. Conditions: 40 μM peptide and 13 μM ThT in 40 mM HEPES-KOH buffer (pH 7.4), supplemented with 150 mM KCl. Error bars indicate SD of 3 measurements.

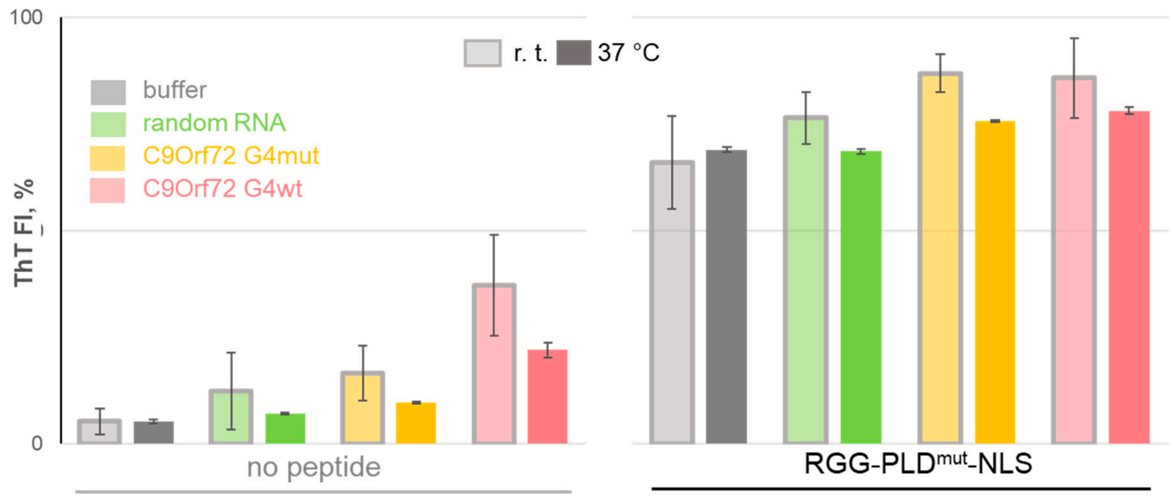

Figure S2. ThT assays after incubation of hnRNP A1 (RGG)-PLD^{mut}-NLS with and without RNA at a room temperature (light bars) and 37 °C (dark bars). Conditions: 40 µM peptide, 13 µM ThT, 0.5 mM DTT, and 0.1 mg/mL random RNA or 20 µM G4 in 40 mM HEPES-KOH buffer (pH 7.4), supplemented with 150 mM KCl.

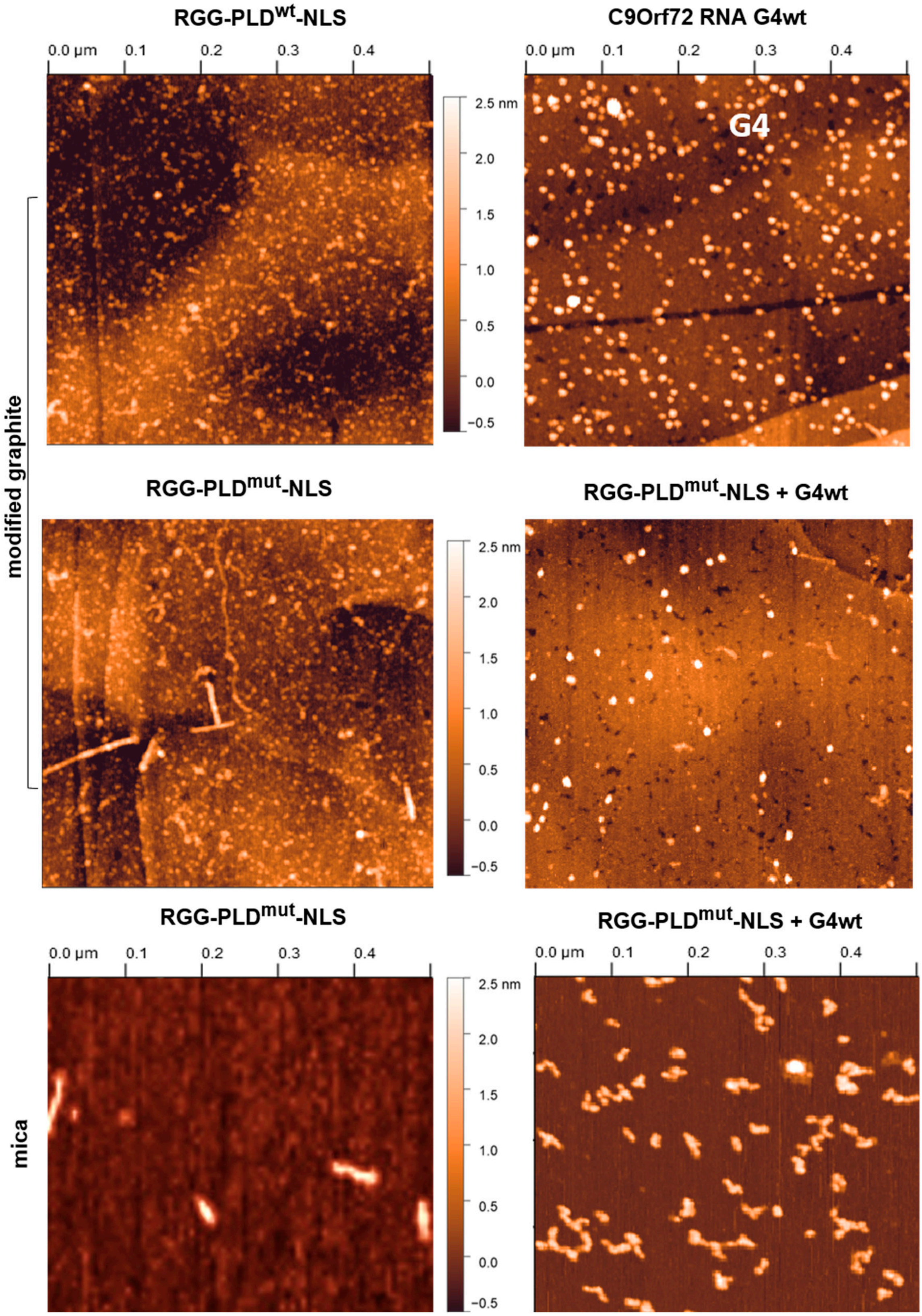

Figure S3. The impact of C9Orf72 RNA G4 on the fibrillation of the mutant hnRNP A1 fragment: large-field atomic force microscopy (AFM) images. Conditions: 40 μM peptide and 20 μM G4 samples were preincubated for 3 h in 40 mM HEPES-KOH buffer (pH 7.4), supplemented with 150 mM KCl and diluted 100x prior to AFM imaging on a modified graphite (a) or mica (b).

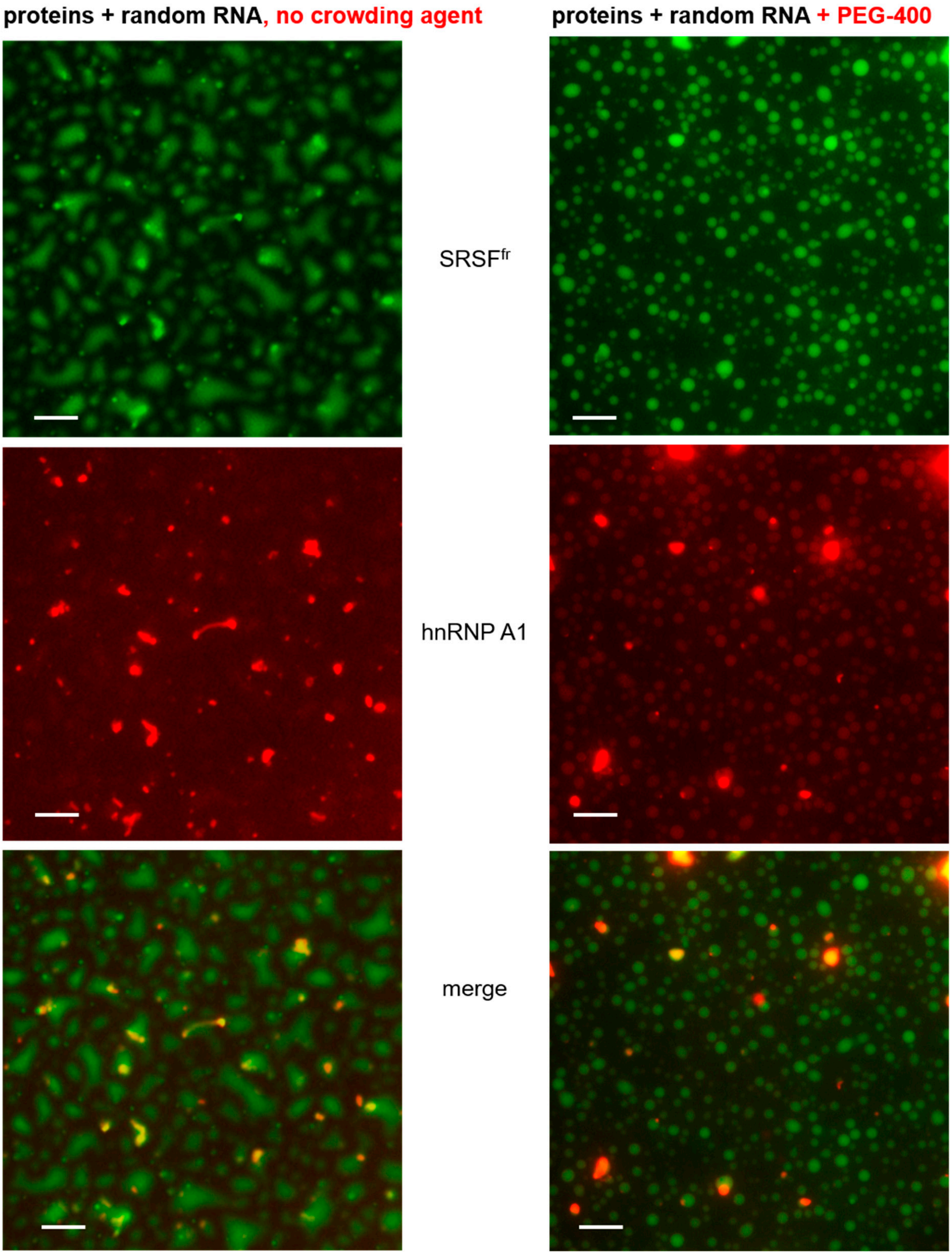

Figure S4. The impact of the crowding agent on the co-separation of hnRNP A1 and SRSF^{fr} in the presence of random RNA: large-field fluorescence microscopy images. Conditions: 6 µM hnRNP A1 (5% RED-labeled),1 mg/mL SRSF^{fr} (5% FITC-labeled), and 3 mg/mL random RNA in PEG-free 40 mM HEPES-KOH buffer (pH 7.4), containing 150 mM KCl (left) or the same buffer supplemented with 10% PEG-400 (right). Scale bar: 50 µm.

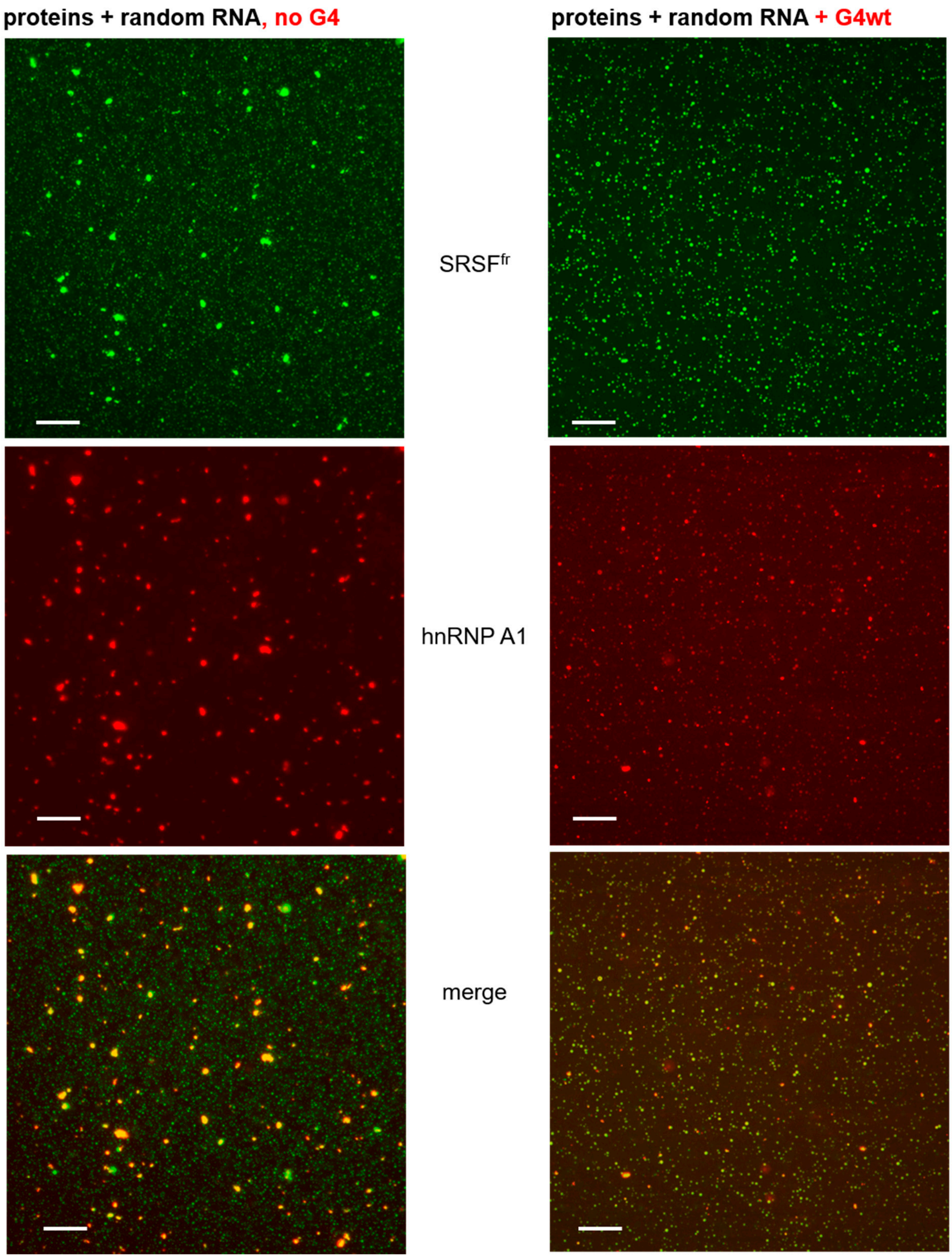

Figure S5. The impact of the G4 on the co-separation of hnRNP A1 and SRSF^{fr} in the presence of random RNA in the crowded environment: large-field fluorescence microscopy images. Conditions: 6 µM hnRNP A1 (5% RED-labeled), 1 mg/mL SRSF^{fr} (5% FITC-labeled), and 3 mg/mL random RNA (left) or 3 mg/mL random RNA with 6 µM C9Orf72 RNA G4wt in 40 mM HEPES-KOH buffer (pH 7.4), supplemented with 150 mM KCl and 10% PEG-400. Scale bar: 10 µm.

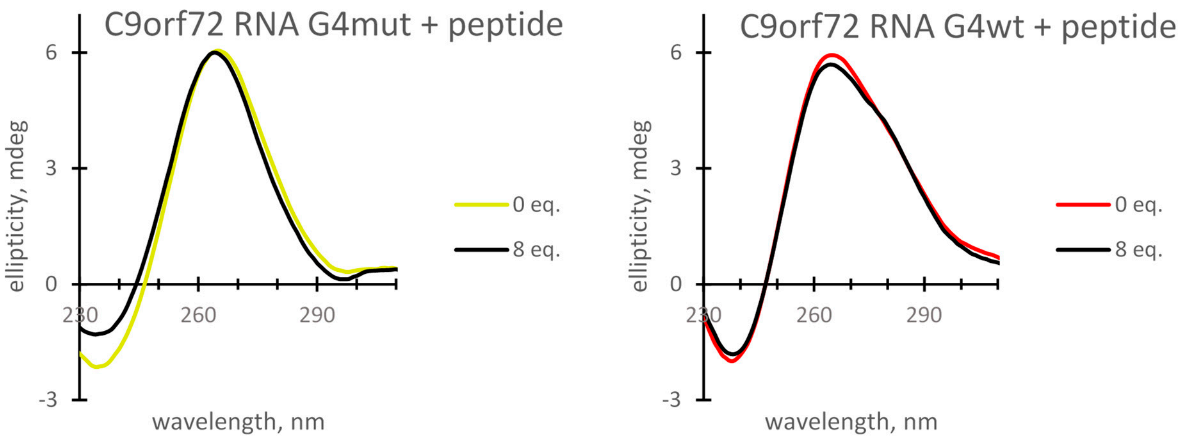

Figure S6. Ellipticity changes upon G4mut (left) or G4wt (right) titration with the peptide PLD^{mut}-NLS. Conditions: 1 μM G4 and 0 or 8 μM peptide in 0.1x HEPES buffer. All spectra were registered at room temperature in 1 cm cuvettes.
